# Equal allocation, demand priority or negotiated allocation? How to allocate water resources in the Tigris and Euphrates River Basin efficiently

**DOI:** 10.1016/j.heliyon.2024.e31458

**Published:** 2024-05-18

**Authors:** Yuntao Bai, Lan Wang, Ruidi Hu, Delong Li

**Affiliations:** a Business School, Shandong Management University, Jinan, 250357, China; b Center of Emergency Management, Chongqing Economic and Social Development Research Institute, Chongqing, 400041, China; c School of Public Administration and Policy, Shandong University of Finance and Economics, Jinan, 250014, China; d School of Business Administration, Inner Mongolia University of Finance and Economics, Hohhot, 010070, China

**Keywords:** Allocation of water resources, Differential games, Social benefit, Two rivers basin, Water-scarce region

## Abstract

The Tigris and Euphrates River Basin is an important water supply, but it suffers from water scarcity. It is necessary to carry out reasonable allocation of water resources in this region. Since water resources issues in this region are of multinational interest, international cooperative distribution efforts are needed. Common water resources allocation modes include equal allocation, demand priority or negotiation allocation. In order to derive the applicable range of various water resources allocation modes, this article constructs three differential game models and compares and analyzes the equilibrium results obtained by the models. Finally, the study shows that when the cost of developing water resources is small and the revenue obtained from developing water resources is large, the water-scarce region can obtain the maximum benefit by adopting the demand priority mode. Otherwise, the water-scarce region can obtain the maximum benefit by adopting the negotiation allocation mode. This study can inform the allocation, strategic interaction and cooperation of dynamic water resources in the two river basins.

## 1 Introduction

### 1.1 Background and research significance

The Tigris and Euphrates River basins, situated in the Middle East, are currently facing a serious water shortage, attributed to a combination of natural and anthropogenic factors. Critical issues contributing to this shortage include reduced river flows and lower groundwater levels, exacerbated by climate change and over-exploitation of resources. Specifically, ongoing climate change is accelerating water evaporation and reducing precipitation, thereby constraining the available water supply. Moreover, frequent droughts have led to a continual decrease in water levels across both river basins.

The region's growing population and rapid economic development have further pressured these limited water resources, sharply increasing the demand. Mismanagement of water resources, characterized by inefficient use and inadequate infrastructure, has also contributed to substantial water waste and loss [1]. Additionally, unresolved disputes over water allocation among countries sharing these river basins worsen the situation, as they hinder effective collaboration and resource sharing [2].

Addressing this pressing issue requires comprehensive and strategic approaches. Middle Eastern countries need to prioritize enhancing water resource management and conservation. This includes investing in modern infrastructure that supports efficient water use in agricultural and industrial sectors, as well as promoting sustainable water practices. It is also critical to restore and protect existing water sources to prevent further depletion.

To resolve conflicts over water sharing, countries must engage in constructive negotiations to devise fair water allocation strategies. These strategies could involve negotiated allocation, where water resources are distributed based on mutual agreements that consider each country's needs and interests. Alternatively, equal allocation could be employed, distributing water evenly according to the population of each region, although this method may need further adjustment to address varying economic needs and development levels more effectively. Another approach is demand priority allocation, where water is directed towards sectors that promise the greatest benefit, such as agriculture, industry, or urban supply, supporting broader economic and social development [3].

In summary, tackling the water shortage in the Tigris and Euphrates River basins requires a combination of improved management, strategic planning, and international cooperation to ensure sustainable and equitable use of this vital resource.

### 1.2 Literature review

Factors influencing the water resources of cross-border rivers have been extensively studied. Piao et al. [4] suggest that climate has a major influence on these resources. Sidibe et al. [5] highlight the critical impact of groundwater mineralization and recharge. Additionally, Saraf and Regulwar [6] focus on the effect of precipitation in the upper Godavari River basin, while Bai et al. [7] discuss the consequences of groundwater over-exploitation. These studies collectively address the impacts of climate, subsurface recharge, precipitation, and over-exploitation on transnational river water resources.

Significant research has also been conducted on water resources management strategies. Khorsandi et al. [8] explore the water carrying capacity evaluation in Iran, and Xu et al. [9] offer an analysis from a visualization technology perspective. Muratoglu et al. [10] investigate management in large hydrological basins of semi-arid regions, and Calvete et al. [11] apply bilevel optimization for resource allocation. Further contributions include Baghanam et al. [12] using system dynamics, Bortoli et al. [13] considering water reuse for conservation, and Moghaddasi et al. [14] developing a stakeholder-based framework for enhancing resilience in arid regions. These studies cover various technologies and theories related to management.

Despite these research efforts, water resource allocation in the Two Rivers Basin presents ongoing challenges, exacerbated by factors such as climate change, demographic growth, and industrial expansion. The growing demand against limited supplies intensifies the supply-demand contradiction, highlighting regional disparities in water use and allocation.

In this context, differential game theory proves to be a robust analytical and mathematical model for understanding decision-makers’ dynamics and strategic choices in the Two Rivers Basin. This theory effectively addresses problems like significant variability in resource allocation. Examples include how limited water resources are utilized and allocated [15], cooperation in allocating transboundary water resources [16], and the distribution of project costs for water resource allocation [17]. Differential game theory can analyze and optimize decision-maker strategies in the Two Rivers Basin's water resource allocation issues. By incorporating difference equations and optimization methods, this model facilitates the determination of optimal strategies while accounting for time and dynamic changes [18,19].

To further detail this analysis, this study utilizes the Hamilton-Jacobi-Bellman (HJB) equation to pinpoint the optimal development amount and maximize benefits across various allocation modes such as equal allocation, demand priority, and negotiation allocation in the Two Rivers Basin.

This paper's innovations lie in its dynamic resource allocation analysis in the two river basins using differential games, allowing for time-impacted strategic adaptations, and its assessment of strategic interactions and cooperation, examining game behavior and the equilibrium between competing and collaborating entities.

By employing differential games, this paper not only provides a nuanced understanding of strategic dynamics and resource optimization in river basin management but also offers practical insights into achieving sustainable and equitable water resource allocation.

## 2 Methodology

### 2.1 Problem description, hypothesis, and variable definition

#### 2.1.1 Problem description

Water allocation in the Tigris and Euphrates basin is significantly influenced by inter-country agreements, each nation's capacity to manage resources, international law principles, and Turkey's control over dams as an upstream country. As of 2023, there is no comprehensive, universally accepted treaty on water allocation despite various bilateral agreements and temporary arrangements established over the years to address the complexities inherited from the substantial national interests and involvement of multiple countries. Historical efforts include a protocol signed in 1990 between Turkey and Syria where Turkey committed to ensuring a minimum flow of 500 m3/s of Euphrates water to Syria at their border. In the 1990s, Syria and Iraq also made some informal agreements concerning the Euphrates, although these did not mature into durable outcomes. Furthermore, tripartite discussions among Turkey, Syria, and Iraq have attempted to address regional water issues but have typically not led to lasting comprehensive agreements [20]. Major Turkish initiatives like the Southeast Anatolia Project (GAP) significantly determine the quantity of water available to downstream nations. However, factors such as climate change, escalating water demands, and geopolitical tensions contribute to the increasing uncertainty of water allocation in the region, often necessitating intricate negotiations and coordination [21].

This study addresses water resource allocation within the Tigris-Euphrates River Basin, prioritizing regions categorized as water-scarce and severely water-scarce based on the universally applied metrics of freshwater availability per capita (cubic meters/person/year): (1) Adequate: over 1,700, (2) Water-scarcity: 1000 to 1,700, (3) Severely water-scarce: 500 to 1,000, and (4) Extreme water shortage: under 500. Focusing on these areas is essential due to their acute water needs, which significantly impact drinking water access, agriculture, energy production, and socio-economic growth, thereby emphasizing the urgency and importance of including these regions in allocation discussions. Water resource allocation is particularly sensitive and contentious in these zones, where equitably distributed water resources are critical for socio-economic development and political stability. Contrarily, regions with abundant water supplies, experiencing less stress, might not be individually spotlighted as key stakeholders. Moreover, water-scarce and severely stressed areas are likely more motivated and willing, through their governments and social organizations, to actively engage in negotiations and agreements on water allocation, unlike more water-abundant regions. However, it's crucial to integrate these classification criteria with considerations of the sustainability of regional water resources, seasonal variations, and disparities in allocation among regions. These aspects are vital in the context of the Tigris and Euphrates basins, which must also account for specific socio-economic conditions, the impacts of climate change, and the complexities of water sharing and conflicts between upstream and downstream nations [22].

Water resources allocation in the Two Rivers Basin is subject to ongoing changes influenced by climate change, population growth, economic development, political instability, and international cooperation. Climate change introduces variability in water supplies through phenomena like drought and irregular rainfall, while global warming accelerates glacier melt, further impacting the basin's water resource stability [23]. Concurrently, the demand for water resources escalates with the increasing population and economic activities, thereby intensifying the pressure on allocation and necessitating more efficient management approaches. Additionally, political instability and inter-regional conflicts over water rights and interests complicate the allocation processes. Importantly, international cooperation through agreements, shared mechanisms, and data exchange on water usage plays a pivotal role in addressing these challenges. Given these dynamics, it is imperative for the countries within the basin to foster collaboration and dialogue towards a more equitable and sustainable water resource allocation strategy.

(1) Equal allocation. Equal water resource allocation is crucial for ensuring fair access for all individuals and regions. To distribute water resources fairly, several methods can be employed. Firstly, equal allocation grants an identical share of water to everyone. Secondly, individual quotas can be set based on factors like population size or geographical area, providing a proportionate share to each entity. Thirdly, timing-based distribution allows equal access during specified periods, such as identical water use durations daily or weekly. Lastly, promoting water conservation can help reduce overall demand and support equitable distribution. However, while equal allocation is appealing, it might not always be the best method due to the need to consider varying demands, sustainability, and efficiency. Thus, a combination of these strategies might be necessary to adapt to specific needs and ensure a practical and fair allocation of water resources.
(2) Demand priority. Allocating water resources based on demand priority is designed to optimize the utilization of limited supplies by directing them toward regions or projects that offer the highest socio-economic benefits [24]. In areas such as the Middle East River Basin, this approach involves several key steps. Initially, an assessment of the demands and socio-economic contributions from different sectors, including agriculture, industry, urban water supply, and ecological protection, is conducted to understand the regional or project-specific needs. Following this, priorities are determined by ranking these regions or projects based on their effectiveness in using water resources and their potential to maximize benefits. Subsequent steps include establishing quotas that reflect these priorities while considering the available water resources. These quotas are set through a collaborative process involving consultation, cooperation, and policy development.
(3) Negotiated allocation mode. Negotiated allocation of water resources plays a pivotal role in resolving disputes and conflicts by involving dialogue, negotiation, and consultation to balance the interests of all parties involved. The process unfolds through several crucial steps. Initially, establishing a formal communication channel is essential to enable open discussions on water allocation issues. Following this, the exchange of vital information concerning water supply, demand, and usage helps all parties to appreciate the needs and limitations of others. Setting common goals is then necessary to align expectations and desired outcomes regarding water allocation. Exploring solutions collectively allows for the consideration of various compromises and the feasibility of different options. Subsequently, negotiation and compromise become the keystones to forging a mutually beneficial agreement. Finally, agreeing on implementation mechanisms ensures that the agreed-upon terms are effectively enacted and monitored over time.

The three modes are interconnected, capable of influencing and intertwining with one another. In fact, the allocation of water resources in the Mesopotamia basin often incorporates the principles of equality and priority of demand, along with the actual negotiation dynamics of all parties, to create a sustainable and widely accepted plan. In practice, water resources allocation frequently adjusts among the three modes to adapt to new demands, shifting political relations, and environmental challenges.

**Fig. 1:**
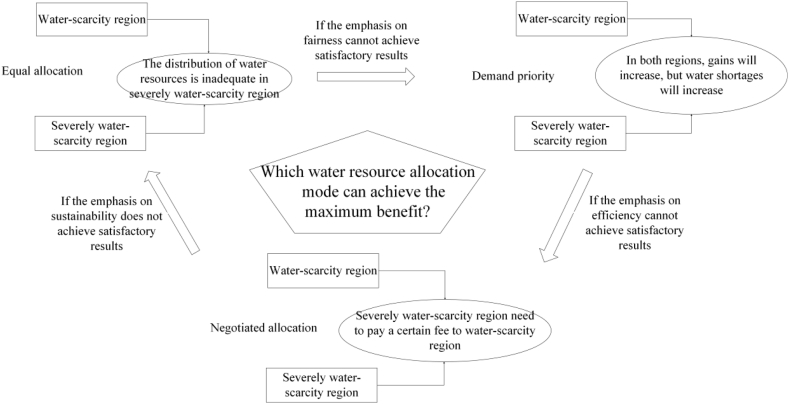
Relationship between three modes of water resources allocation.

The relationship between three modes of water resources allocation is shown in Fig. 1.

#### 2.1.2 Hypothesis

Hypothesis 1 Equal allocation will lead to under-allocation of water resources in region with severe water shortage

In the Two Rivers Basin, the challenge of efficiently distributing limited water resources is exacerbated by geographic disparities and varying degrees of shortages, especially severe in regions like Iraq's lower Euphrates. The Euphrates and Tigris, crucial rivers in the area, are shared among several countries, necessitating cooperative management. An approach focusing solely on equal water distribution overlooks the critical needs of those areas facing dire shortages, potentially aggravating their struggles and impacting social stability and development [25]. Therefore, a more nuanced strategy that considers differences in supply and demand, prioritizes environmental protection, and aims for efficiency improvements is essential.

Hypothesis 2 The demand for water in a region includes both basic and additional needs.

In the Middle East, particularly in Mesopotamia, water scarcity has hit critical levels, presenting enormous challenges for the region's demands, which span from basic needs to additional requirements due to economic development. Basic demands are centered around agricultural irrigation, which is the region's largest water consumer, driven by its pivotal role in the economy amidst an arid climate, low rainfall, and high temperatures. Drinking water is another fundamental need, with the growing population and urbanization intensifying the pressure on resources needed for purification, transmission, and allocation. Furthermore, the importance of water for environmental and ecological purposes, such as ecosystem protection and preventing resource over-exploitation, underscores the complex challenge of balancing economic growth with ecological sustainability in arid areas like the Two Rivers Basin. This multifaceted dilemma requires innovative management strategies to ensure all needs are met without compromising the region's ecological integrity or the well-being of its inhabitants.

Hypothesis 3 Under the negotiated allocation mode, the severe water shortage area needs to pay a certain amount of money to the water shortage area.

Managing the Two Rivers Basin's water resources efficiently requires a strategic approach that includes a negotiated allocation model with a component of fee payment to water-scarce regions. This fee serves as compensation for the insufficient water allocation encountered by these areas, facilitating several critical objectives. Firstly, it helps improve the supply-demand balance by transferring financial resources from water-abundant regions to those critically under-supplied, thereby enhancing their water availability and promoting overall equilibrium. Secondly, the fee incentivizes water conservation and fosters efficient resource use in areas facing shortages, thus mitigating water stress and reinforcing a commitment to sustainable practices throughout the region. Thirdly, the implementation of fee payments can bolster inter-regional cooperation, stimulate collaborative water management efforts, and encourage the development of innovative solutions through transnational agreements and joint technological initiatives.

#### 2.1.3 Variable definition

When constructing the differential game model in this article, many parameters and variables are designed. These parameters and variables are defined as shown in Table 1.

**Table 1:** The main definition of variables and parameters in this article.

variables and parameters	specific meaning
*Y =* {*A,R,N*}	three modes of water resources allocation (equal allocation, demand priority, negotiated allocation)
independent variable	
*G*_*Y*1_(*t*)	the amount of water resources allocated to the water-scarcity region under allocation mode *Y*
*G*_*Y*2_(*t*)	the amount of water resources allocated to the severely water-scarcity region under allocation mode *Y*
*x*_*Y*1_(*t*)	the water-scarce region’ water shortage degree under the control mode *Y*
*x*_*Y*2_(*t*)	the severely water-scarce region’ water shortage degree under the control mode *Y*
parameter	
*ρ*	the discount rate that occurs over time, 0 ≤ *ρ* ≤ 1
*δ*	self-resilience of water resources, *δ* > 0
*b*_1_, *b*_2_	benefits of a unit water allocation to water-scarce or severely water-scarce region, *b*_1_, *b*_2_ > 0
*c*_1_,*c*_2_	the cost of developing a unit of water resources in water-scarce or severely water-scarce region, *c*_1_, *c*_2_ > 0
*l*	long-term effects of water scarcity, *l* > 0
*q*_*I*_	water resources demand in severely water-scarce region, *q*_*I*_ > 0
*a*_2_	the impact of a unit water shortage in the severely water-scarce region, *a*_2_>0
*f*_1_,*f*_2_	impact of extraction per unit on water shortage, *f*_1_,*f*_2_ > 0
*b*_*R*_	additional revenue from allocating water resources according to demand, *b*_*R*_ > 0
*f*	water shortages exacerbated by the need to allocate water resources, *f* > 0
*F*_*N*_	the amount of money that the severely water-scarce region pays to the water-scarcity region under the negotiated allocation mode, *F*_*N*_ > 0
function	
*J*_*Y*1_(*t*)	the social welfare function of water-scarce region under the allocation mode *Y*
*J*_*Y*2_(*t*)	the social welfare function of severely water-scarce region under the allocation mode *Y*
*V*_*Y*1_(*t*)	the social benefits of water-scarce region under the allocation mode *Y*
*V*_*Y*2_(*t*)	the social benefits of severely water-scarce region under the allocation mode *Y*

### 2.2 Differential game of three allocation modes

Differential games is a branch of game theory that studies game models in which players make decisions in continuous time. Unlike discrete-time games, which take place at separate intervals, differential games operate within a continuous time framework, allowing players to adjust their strategies to meet optimization goals. In differential games, the strategies players use may be represented as continuous functions because they make decisions over an ongoing time period. Players aim to select the optimal strategy to maximize their utility function. The utility function can be a player's profit, benefit, or other objective. Differential games typically involve systems of differential equations, where players' strategies influence the system's state development. Players need to adjust their strategies in time to maximize their utility according to the dynamic evolution of the system. Research methods in differential games often include control theory and differential equations [26]. In summary, differential games examine models where players make continuous-time decisions to pursue optimization objectives through strategy refinement. The primary research methods are differential equations and control theory.

Generally, when regions compete for water resources, prioritizing the fundamental allocation of water is essential to meet basic needs, including agricultural irrigation that directly impacts livelihoods. However, with Mesopotamia's water shortages, the equal allocation approach prioritizes equity over specific needs. If the regions of the Two Rivers Basin adopt the mode of equal allocation to allocate water resources, then the social welfare function of the water-scarce region and the severely water-scarce region can be expressed as:

(1) $$J_{A1}=\int_{0}^{\infty }[b_{1}G_{A1}\left(t\right)-\frac{c_{1}}{2}G_{A1}^{2}\left(t\right)+lx_{A1}\left(t\right)]\;e^{-\rho t}dt$$

(2) $$J_{A2}=\int_{0}^{\infty }[b_{2}G_{A2}\left(t\right)-\frac{c_{2}}{2}G_{A2}^{2}\left(t\right)-\left(q_{I}-G_{A2}\left(t\right)\right)a_{2}+lx_{A2}\left(t\right)]\;e^{-\rho t}dt$$

In the above formula, $b_{1}G_{A1}\left(t\right)$ represents the income gained by water resources development and utilization in water-scarce region under the average allocation mode. $\frac{c_{1}}{2}G_{A1}^{2}\left(t\right)$ represents the cost of water resources development and utilization in water-scarce region under the average allocation mode. $lx_{A1}\left(t\right)$ represents the long-term impact of water resources shortage on water-scarce region under the average allocation mode. $b_{2}G_{A2}\left(t\right)$ represents the income gained by water resources development and utilization in severe water-scarce region under the average allocation mode. $\frac{c_{2}}{2}G_{A2}^{2}\left(t\right)$ represents the cost of water resources development and utilization in severe water-scarce region under the average allocation mode. $\left(q_{I}-G_{A2}\left(t\right)\right)a_{2}$ represents the short-term impact of water resources shortage on severe water-scarce region under the average allocation mode. $lx_{A2}\left(t\right)$ represents the long-term impact of water resources shortage on severe water-scarce region under the average allocation mode.

Under the mode of equal allocation of water resources, the changes of water shortage degree in water-scarce region and severely water-scarce region can be expressed as:

(3) $${\dot{x}}_{A1}\left(t\right)=-f_{1}G_{A1}\left(t\right)+\delta x_{A1}\left(t\right)$$

(4) $${\dot{x}}_{A2}\left(t\right)=-f_{2}G_{A2}\left(t\right)+\delta x_{A2}\left(t\right)$$

In the above formula, $f_{1}G_{A1}\left(t\right)$ represents the negative impact of water resources development in water-scarce region while water resources shortage. $f_{2}G_{A2}\left(t\right)$ represents the negative impact of water resources development in severely water-scarce region while water resources shortage. $\delta x_{A1}\left(t\right)$ represents the self-recovery of water resources in water-scarce region. $\delta x_{A2}\left(t\right)$ represents the self-recovery of water resources in severely water-scarce region.

Under the demand priority mode, not only the basic demand for water resources (such as agricultural irrigation and basic life), but also the additional demand for water resources (such as the development of secondary and tertiary industries) should be considered. This is different from the equal allocation mode. If the regions of the Two Rivers Basin adopt the mode of demand priority to allocate water resources, then the social welfare function of the water-scarce region and the severely water-scarce region can be expressed as:

(5) $$J_{R1}=\int_{0}^{\infty }[\left(b_{1}+b_{R}\right)G_{R1}\left(t\right)-\frac{c_{1}}{2}G_{R1}^{2}\left(t\right)+lx_{R1}\left(t\right)]\;e^{-\rho t}dt$$

(6) $$J_{R2}=\int_{0}^{\infty }[\left(b_{2}+b_{R}\right)G_{R2}\left(t\right)-\frac{c_{2}}{2}G_{R2}^{2}\left(t\right)+lx_{R2}\left(t\right)]\;e^{-\rho t}dt$$

In the above formula, $b_{1}G_{R1}\left(t\right)$ represents the benefits of developing and utilizing water resources to meet basic needs in water-scarce region under the demand priority mode. These basic needs include water for daily use, irrigation of crops, etc. $b_{R}G_{R1}\left(t\right)$ represents the additional benefits of developing and utilizing water resources in water-scarce region under the demand priority mode. These additional benefits include those from industrial production, the development of tourism and other secondary and tertiary industries. $\frac{c_{1}}{2}G_{R1}^{2}\left(t\right)$ represents the costs of developing and utilizing water resources in water-scarce region under the demand priority mode. $lx_{R1}\left(t\right)$ represents the long-term impact of water shortage on water-scarce region under the demand priority mode. $b_{2}G_{R2}\left(t\right)$ represents the benefits of developing and utilizing water resources in severely water-scarce region under the demand priority mode. $b_{R}G_{R2}\left(t\right)$ represents the additional benefits of developing and utilizing water resources in severely water-scarce region under the demand priority mode. $\frac{c_{2}}{2}G_{R2}^{2}\left(t\right)$ represents the costs of developing and utilizing water resources in severely water-scarce region under the demand priority mode. $lx_{R2}\left(t\right)$ represents the long-term impact of water shortage on severely water-scarce region under the demand priority mode.

Under the mode of demand priority of water resources, the changes of water shortage degree in water-scarce region and severely water-scarce region can be expressed as:

(7) $${\dot{x}}_{R1}\left(t\right)=-\left(f+f_{1}\right)G_{R1}\left(t\right)+\delta x_{R1}\left(t\right)$$

(8) $${\dot{x}}_{R2}\left(t\right)=-\left(f+f_{2}\right)G_{R2}\left(t\right)+\delta x_{R2}\left(t\right)$$

In the above formula, $f_{1}G_{R1}\left(t\right)$ represents the negative impact of water resources development in water-scarce region. $f_{2}G_{R2}\left(t\right)$ represents the negative impact of water resources development in severely water-scarce region. $fG_{R1}\left(t\right)$ represents the loss caused by excessive water resources development in water-scarce region. $fG_{R2}\left(t\right)$ represents the loss caused by excessive water resources development in severely water-scarce region. $\delta x_{R1}\left(t\right)$ represents the self-recovery of water resources in water-scarce region. $\delta x_{R2}\left(t\right)$ represents the self-recovery of water resources in severely water-scarce region.

If the regions of the Two Rivers Basin adopt the mode of negotiated allocation to allocate water resources, then the social welfare function of the water-scarce region and the severely water-scarce region can be expressed as:

(9) $$J_{N1}=\int_{0}^{\infty }[b_{1}G_{N1}\left(t\right)-\frac{c_{1}}{2}G_{N1}^{2}\left(t\right)+F_{N}+lx_{N1}\left(t\right)]\;e^{-\rho t}dt$$

(10) $$J_{N2}=\int_{0}^{\infty }[b_{2}G_{N2}\left(t\right)-\frac{c_{2}}{2}G_{N2}^{2}\left(t\right)-F_{N}+lx_{N2}\left(t\right)]\;e^{-\rho t}dt$$

In the above formula, $b_{1}G_{N1}\left(t\right)$ represents the benefits of developing and utilizing water resources in water-scarce region under the negotiated allocation mode. $\frac{c_{1}}{2}G_{N1}^{2}\left(t\right)$ represents the costs of developing and utilizing water resources in water-scarce region under the negotiated allocation mode. $lx_{N1}\left(t\right)$ represents the long-term impact of water shortage on water-scarce region under the negotiated allocation mode. $b_{2}G_{N2}\left(t\right)$ represents the benefits of developing and utilizing water resources in severely water-scarce region under the negotiated allocation mode. $\frac{c_{2}}{2}G_{N2}^{2}\left(t\right)$ represents the costs of developing and utilizing water resources in severely water-scarce region under the negotiated allocation mode. $lx_{N2}\left(t\right)$ represents the long-term impact of water shortage on severely water-scarce region under the negotiated allocation mode.

Under the mode of negotiated allocation of water resources, the changes of water shortage degree in water-scarce region and severely water-scarce region can be expressed as:

(11) $${\dot{x}}_{N1}\left(t\right)=-f_{1}G_{N1}\left(t\right)+\delta x_{N1}\left(t\right)$$

(12) $${\dot{x}}_{N2}\left(t\right)=-f_{2}G_{N2}\left(t\right)+\delta x_{N2}\left(t\right)$$

In the above formula, $f_{1}G_{N1}\left(t\right)$ represents the negative impact of water resources development in water-scarce region. $f_{2}G_{N2}\left(t\right)$ represents the negative impact of water resources development in severely water-scarce region. $\delta x_{N1}\left(t\right)$ represents the self-recovery of water resources in water-scarce region. $\delta x_{N2}\left(t\right)$ represents the self-recovery of water resources in severely water-scarce region.

## 3 Results

In the differential game, water resources development decisions in water-scarce and severely water-scarce region are not only affected by control variables and parameters, but also change with time. In order to better calculate the control amount and social benefit, the HJB formula is adopted. HJB formula is a partial differential equation, which is the core of optimal control.

### 3.1 HJB formula

In differential games, the HJB equation plays a central role, which is used to describe optimal strategies in the context of game theory. Differential games are games in which the strategies of the players (or "players" of the game) develop in continuous time and continuously affect the state of a dynamic system. Similar to its role in single control problems, the HJB equation in differential games describes the value function's evolution, the system's state, and how players minimize or maximize their own cost function by choosing the optimal strategy [27]. Within the game, each player has their own value function, aiming to optimize their payment. For example, in a two-player differential game, if we assume two players each aim to maximize their value function, we end up with two simultaneous HJB equations, one for each player. Each equation takes into account the optimal strategies of other actors, thus forming a policy-dependent system [28].

Under the mode of equal allocation, the HJB equation of the social welfare function of water-scarce region and severely water-scarce region are:

(13) $$\rho V_{A1}=\max_{G_{A1}\left(t\right)}\{[b_{1}G_{A1}\left(t\right)-\frac{c_{1}}{2}G_{A1}^{2}\left(t\right)+lx_{A1}\left(t\right)]+\frac{\partial V_{A1}}{\partial x_{A1}}\left[-f_{1}G_{A1}\left(t\right)+\delta x_{A1}\left(t\right)\right]\}$$

(14) $$\rho V_{A2}=\max_{G_{A2}\left(t\right)}\{[b_{2}G_{A2}\left(t\right)-\frac{c_{2}}{2}G_{A2}^{2}\left(t\right)-\left(q_{I}-G_{A2}\left(t\right)\right)a_{2}+lx_{A2}\left(t\right)]+\frac{\partial V_{A2}}{\partial x_{A2}}\left[-f_{2}G_{A2}\left(t\right)+\delta x_{A2}\left(t\right)\right]\}$$

Under the mode of demand priority, the HJB equation of the social welfare function of water-scarce region and severely water-scarce region are:

(15) $$\rho V_{R1}=\max_{G_{R1}\left(t\right)}\{[\left(b_{1}+b_{R}\right)G_{R1}\left(t\right)-\frac{c_{1}}{2}G_{R1}^{2}\left(t\right)+lx_{A1}\left(t\right)]+\frac{\partial V_{R1}}{\partial x_{R1}}\left[-\left(f+f_{1}\right)G_{R1}\left(t\right)+\delta x_{R1}\left(t\right)\right]\}$$

(16) $$\rho V_{R2}=\max_{G_{R2}\left(t\right)}\{[\left(b_{2}+b_{R}\right)G_{R2}\left(t\right)-\frac{c_{2}}{2}G_{R2}^{2}\left(t\right)+lx_{A2}\left(t\right)]+\frac{\partial V_{R2}}{\partial x_{R2}}\left[-\left(f+f_{2}\right)G_{R2}\left(t\right)+\delta x_{R2}\left(t\right)\right]\}$$

Under the mode of negotiated allocation, the HJB equation of the social welfare function of water-scarce region and severely water-scarce region are:

(17) $$\rho V_{N1}=\max_{G_{N1}\left(t\right)}\{[b_{1}G_{N1}\left(t\right)-\frac{c_{1}}{2}G_{N1}^{2}\left(t\right)+F_{N}+lx_{N1}\left(t\right)]+\frac{\partial V_{N1}}{\partial x_{N1}}\left[-f_{1}G_{N1}\left(t\right)+\delta x_{N1}\left(t\right)\right]\}$$

(18) $$\rho V_{N2}=\max_{G_{N2}\left(t\right)}\{[b_{2}G_{N2}\left(t\right)-\frac{c_{2}}{2}G_{N2}^{2}\left(t\right)-F_{N}+lx_{N2}\left(t\right)]+\frac{\partial V_{N2}}{\partial x_{N2}}\left[-f_{2}G_{N2}\left(t\right)+\delta x_{N2}\left(t\right)\right]\}$$

### 3.2 Result of equilibrium

Proposition 1 Under the mode of equal allocation, the water resources development amount and social benefits of the water-scarce region and severely water-scarce region are respectively (the specific solving procedure is shown in Appendix 1):

(19) $$G_{A1}^{*}\left(t\right)=\frac{b_{1}-\frac{l}{\rho -\delta }f_{1}}{c_{1}}$$

(20) $$G_{A2}^{*}\left(t\right)=\frac{b_{2}+a_{2}-\frac{l}{\rho -\delta }f_{2}}{c_{2}}$$

(21) $$V_{A1}^{*}=\frac{l}{\rho -\delta }x_{A1}+\frac{1}{\rho }b_{1}\frac{b_{1}-\frac{l}{\rho -\delta }f_{1}}{c_{1}}-\frac{c_{1}}{2}\frac{1}{\rho }{\left(\frac{b_{1}-\frac{l}{\rho -\delta }f_{1}}{c_{1}}\right)}^{2}-\frac{1}{\rho }\frac{l}{\rho -\delta }f_{1}\frac{b_{1}-\frac{l}{\rho -\delta }f_{1}}{c_{1}}$$

(22) $$V_{A2}^{*}=\frac{l}{\rho -\delta }x_{A2}+\frac{1}{\rho }b_{2}\frac{b_{2}+a_{2}-\frac{\partial V_{A2}}{\partial x_{A2}}f_{2}}{c_{2}}-\frac{c_{2}}{2}\frac{1}{\rho }{\left(\frac{b_{2}+a_{2}-\frac{\partial V_{A2}}{\partial x_{A2}}f_{2}}{c_{2}}\right)}^{2}-\frac{1}{\rho }\left(q_{I}-\frac{b_{2}+a_{2}-\frac{\partial V_{A2}}{\partial x_{A2}}f_{2}}{c_{2}}\right)a_{2}-\frac{\partial V_{A2}}{\partial x_{A2}}\frac{1}{\rho }f_{2}\frac{b_{2}+a_{2}-\frac{\partial V_{A2}}{\partial x_{A2}}f_{2}}{c_{2}}$$

Conclusion 1 Under the average allocation mode, the greater the impact of water exploitation on water shortage, the smaller the water resources allocated to a region.

Proposition 2 Under the mode of demand priority, the water resources development amount and social benefits of the water-scarce region and severely water-scarce region are respectively (the specific solving procedure is shown in Appendix 2):

(23) $$G_{R1}^{*}\left(t\right)=\frac{b_{1}+b_{R}-\frac{l}{\rho -\delta }\left(f_{1}+f\right)}{c_{1}}$$

(24) $$G_{R2}^{*}\left(t\right)=\frac{b_{2}+b_{R}-\frac{l}{\rho -\delta }\left(f_{2}+f\right)}{c_{2}}$$

(25) $$V_{R1}^{*}=\frac{l}{\rho -\delta }x_{R1}+\frac{1}{\rho }\left(b_{1}+b_{R}\right)\frac{b_{1}+b_{R}-\frac{l}{\rho -\delta }\left(f_{1}+f\right)}{c_{1}}-\frac{c_{1}}{2}\frac{1}{\rho }{\left[\frac{b_{1}+b_{R}-\frac{l}{\rho -\delta }\left(f_{1}+f\right)}{c_{1}}\right]}^{2}-\frac{l}{\rho -\delta }\frac{1}{\rho }\left(f+f_{1}\right)\frac{b_{1}+b_{R}-\frac{l}{\rho -\delta }\left(f_{1}+f\right)}{c_{1}}$$

(26) $$V_{R2}^{*}=\frac{l}{\rho -\delta }x_{R2}+\frac{1}{\rho }\left(b_{2}+b_{R}\right)\frac{b_{2}+b_{R}-\frac{l}{\rho -\delta }\left(f_{2}+f\right)}{c_{2}}-\frac{c_{2}}{2}\frac{1}{\rho }{\left[\frac{b_{2}+b_{R}-\frac{l}{\rho -\delta }\left(f_{2}+f\right)}{c_{2}}\right]}^{2}-\frac{l}{\rho -\delta }\frac{1}{\rho }\left(f+f_{2}\right)\frac{b_{2}+b_{R}-\frac{l}{\rho -\delta }\left(f_{2}+f\right)}{c_{2}}$$

Conclusion 2 Under the demand priority allocation mode, the greater the water shortage degree emphasized by the allocation method, the smaller the water resources allocated to a region.

Proposition 3 Under the mode of negotiated allocation, the water resources development amount and social benefits of the water-scarce region and severely water-scarce region are respectively (the specific solving procedure is shown in Appendix 3):

(27) $$G_{N1}^{*}\left(t\right)=\frac{b_{1}-\frac{l}{\rho -\delta }f_{1}}{c_{1}}$$

(28) $$G_{N2}^{*}\left(t\right)=\frac{b_{2}-\frac{l}{\rho -\delta }f_{2}}{c_{2}}$$

(29) $$V_{N1}^{*}=\frac{l}{\rho -\delta }x_{N1}+\frac{1}{\rho }b_{1}\frac{b_{1}-\frac{l}{\rho -\delta }f_{1}}{c_{1}}-\frac{c_{1}}{2}\frac{1}{\rho }{\left(\frac{b_{1}-\frac{l}{\rho -\delta }f_{1}}{c_{1}}\right)}^{2}+\frac{1}{\rho }F_{N}-\frac{1}{\rho }\frac{l}{\rho -\delta }f_{1}\frac{b_{1}-\frac{l}{\rho -\delta }f_{1}}{c_{1}}$$

(30) $$V_{N2}^{*}=\frac{l}{\rho -\delta }x_{N2}+\frac{1}{\rho }b_{2}\frac{b_{2}-\frac{l}{\rho -\delta }f_{2}}{c_{2}}-\frac{c_{2}}{2}\frac{1}{\rho }{\left(\frac{b_{2}-\frac{l}{\rho -\delta }f_{2}}{c_{2}}\right)}^{2}-\frac{1}{\rho }F_{N}-\frac{l}{\rho -\delta }\frac{1}{\rho }f_{2}\frac{b_{2}-\frac{l}{\rho -\delta }f_{2}}{c_{2}}$$

Conclusion 3 Under the negotiated allocation mode, the greater the impact of unit mining amount on water shortage degree, the smaller the water resources allocated to a region.

### 3.3 Numerical analysis

In order to describe the changes of social utility in water scarcity and severe water scarcity in the process of water resources allocation in more detail, this article adopts the numerical analysis method.

The discount rate *ρ* that occurs over time is 0.9. This is mainly because the discount factor can reflect the uncertainty and potential risks of future cash flows. A lower discount factor (e.g. 0.9) means a higher risk premium for future cash flows, i.e. investors or decision makers require higher returns to compensate for the higher risks they take [29].

The recovery rate of water resources in the Middle East Mesopotamia Basin is slow, mainly due to climate change, high water resources development intensity, water cycle imbalance and other factors. In order to reflect this slow recovery rate, this paper assumes that self-resilience *δ* of water resources is 0.1.

In the Mesopotamia, the urgency of water resources management stems from two types of problems: acute shortages in the short term and long-term water scarcity. In acute water scarcity areas The impact of water scarcity per unit includes the following aspects. First, immediate impact. Acute water scarcity immediately affects the daily lives of residents, agricultural production, and industry, potentially triggering water supply crises, social instability, and economic losses. Second, urgency. Severe water scarcity can fail to meet basic human needs like drinking, hygiene, and food production. Such cases demand immediate responses due to direct consequences. Third, humanitarian crisis. Extreme water scarcity may lead to drought, famine and the spread of diseases, posing a direct threat to the health and lives of the population. Fourth, economic losses. Water scarcity restricts crop growth and industrial capacity, directly impacting the economy. Extreme water scarcity may aggravate social conflicts and conflicts, especially in areas where water demand exceeds water supply [20]. Although serious, the long-term impacts of water scarcity are progressive and can be mitigated or adapted through continuous planning and management. Effective long-term water management strategies can even prevent these impacts. First, coping strategies. Governments and communities can use the time during long-term resource scarcity to develop water-saving measures, alternative water sources, and promote conservation policies. Second, structural adjustments. Adapting to long-term water scarcity involves gradual structural adjustments such as planting drought-tolerant crops, improving water infrastructure, and educating consumers. Third, technological innovation. New technologies like water recycling, rainwater harvesting, and seawater desalination have been developed in response to long-term water scarcity. Issues of urgent water scarcity typically receive more attention and resources, making “the per unit impact in a severely water-scarce region” appear more serious than “the long-term impacts of water scarcity”. Long-term effects *l* of water scarcity is 1. The impact *a*_2_ of a unit water shortage in the severely water-scarce region is 2. If the effects of extracting water are equally divided between short-term and long-term effects, then the paper assumes that impact *f*_1_,*f*_2_ of extraction per unit on water shortage is 1.5. If water resources can be allocated reasonably, the shortage caused by water allocation is less than that caused by water exploitation. Therefore, this paper assumes that water shortages *f* exacerbated by the need to allocate water resources is 1.

Meanwhile, in the case of water scarcity, rational allocation of water resources can significantly reduce the adverse impacts caused by inadequate water supply [30]. The marginal value of water tends to be very high in areas experiencing severe water scarcity. This implies that each additional unit of water supplied results in significant benefits (or avoidance of losses) due to the high demand elasticity in these areas. Therefore,“the impact of a unit water shortage in the severely water-scarce region”is equal to “additional revenue from allocating water resources according to demand ”. Therefore, the additional revenue *b*_*R*_ from allocating water resources according to demand is 2.

In addition, the water shortage problem in the Mesopotamia is very serious [20]. Therefore, this paper hypothesizes that water resources demand *q*_*I*_ in severely water-scarce region is 5. The amount *F*_*N*_ of money that the severely water-scarce region pays to the water-scarcity region under the negotiated allocation mode is 3.

When the cost *c*_1_,*c*_2_ of developing a unit of water resources in water-scarce or severely water-scarce region is 2, this article can calculate the social benefits of water-scarce region:

(31) $$V_{A1}^{*}=1.25+0.278\times {\left(b_{1}-1.875\right)}^{2}$$

(32) $$V_{R1}^{*}=1.25+0.278\times {\left(b_{1}-1.125\right)}^{2}$$

(33) $$V_{N1}^{*}=4.583+0.278\times {\left(b_{1}-1.875\right)}^{2}$$

**Fig. 2:**
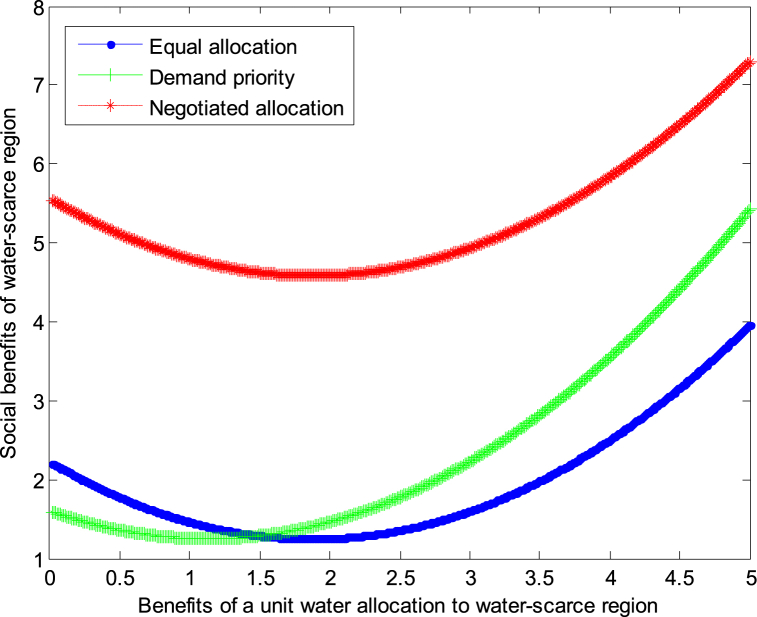
Impact of benefit of water-scarce region on social welfare.

**Fig. 3:**
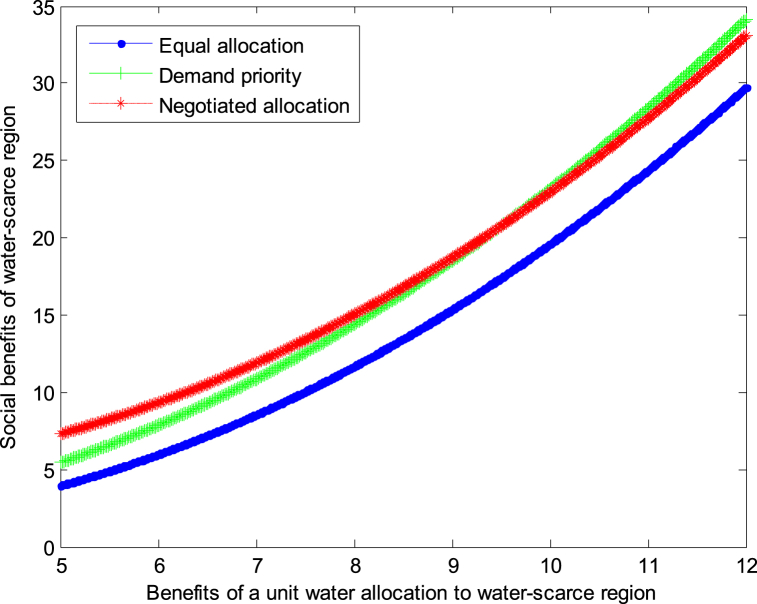
Impact of benefit of water-scarce region on social welfare.

The following graph (named Fig. 2, Fig. 3) can also be produced.

Conclusion 4 When the cost of developing water resources is small and the benefits obtained from developing water resources are small, the negotiated allocation mode can achieve the maximum benefit in water-scarce region. When the cost of developing water resources is small and the benefits obtained from developing water resources are large, the demand priority mode can achieve the maximum benefit in water-scarce region.

When the cost *c*_1_,*c*_2_ of developing a unit of water resources in water-scarce or severely water-scarce region is 4, this article can calculate the social benefits of water-scarce region:

(34) $$V_{A1}^{*}=1.25+0.139\times {\left(b_{1}-1.875\right)}^{2}$$

(35) $$V_{R1}^{*}=1.25+0.139\times {\left(b_{1}-1.125\right)}^{2}$$

(36) $$V_{N1}^{*}=4.583+0.139\times {\left(b_{1}-1.875\right)}^{2}$$

**Fig. 4:**
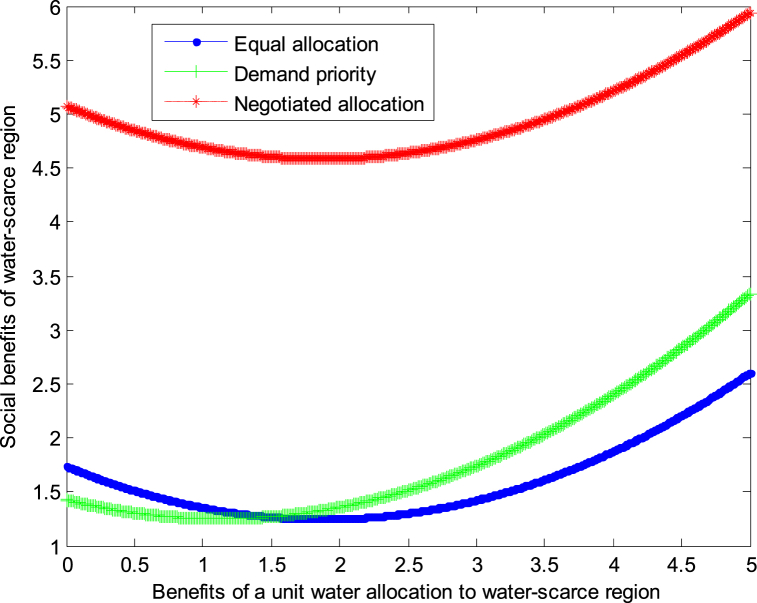
Impact of benefit of water-scarce region on social welfare.

**Fig. 5:**
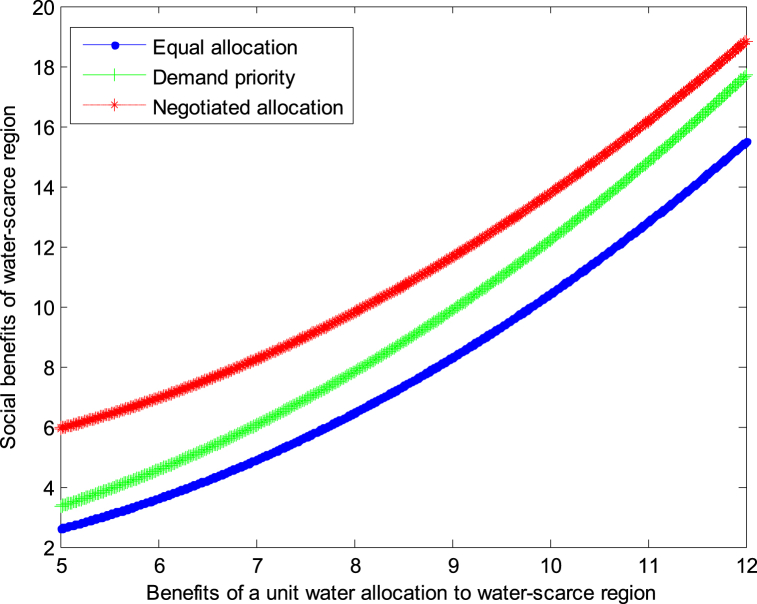
Impact of benefit of water-scarce region on social welfare.

The following graph (named Fig. 4, Fig. 5) can also be produced.

Conclusion 5 When the cost of developing water resources is high, the negotiated allocation mode can obtain the maximum benefit in water-scarce region.

When the cost *c*_1_,*c*_2_ of developing a unit of water resources in water-scarce or severely water-scarce region is 2, this article can calculate the social benefits of severely water-scarce region:

(37) $$V_{A2}^{*}=-9.722+1.111b_{2}+0.278\times {\left(b_{2}+0.125\right)}^{2}$$

(38) $$V_{R2}^{*}=1.25+0.278\times {\left(b_{2}-1.125\right)}^{2}$$

(39) $$V_{N2}^{*}=-2.083+0.278\times {\left(b_{2}-1.875\right)}^{2}$$

**Fig. 6:**
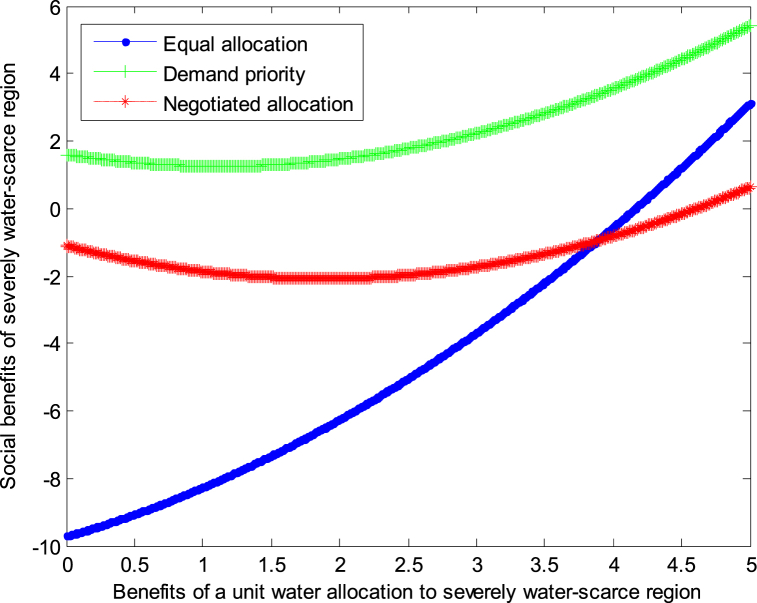
Impact of benefit of severely water-scarce region on social welfare.

**Fig. 7:**
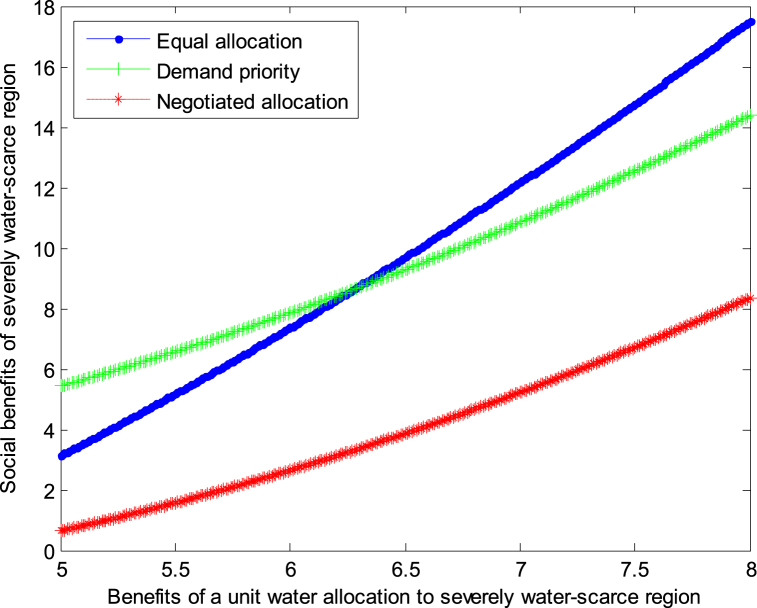
Impact of benefit of severely water-scarce region on social welfare.

The following graph (named Fig. 6, Fig. 7) can also be produced.

When the cost *c*_1_,*c*_2_ of developing a unit of water resources in water-scarce or severely water-scarce region is 4, this article can calculate the social benefits of severely water-scarce region:

(40) $$V_{A2}^{*}=-9.792+0.556b_{2}+0.139\times {\left(b_{2}+0.125\right)}^{2}$$

(41) $$V_{R2}^{*}=1.25+0.139\times {\left(b_{2}-1.125\right)}^{2}$$

(42) $$V_{N2}^{*}=-2.083+0.139\times {\left(b_{2}-1.875\right)}^{2}$$

**Fig. 8:**
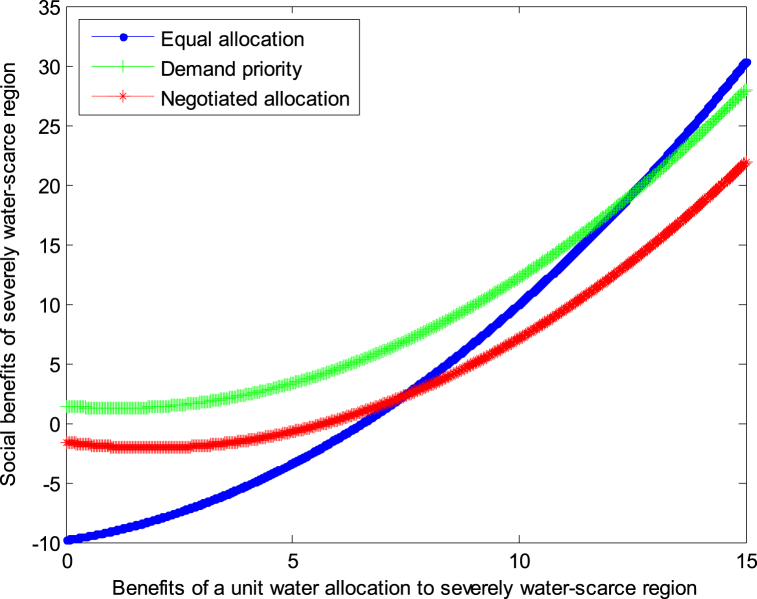
Impact of benefit of severely water-scarce region on social welfare.

The following graph (named Fig. 8) can also be produced.

Conclusion 6 : No matter how the cost of water resources development changes, when the benefits from water resources development are small, the demand priority mode can be adopted in severely water-scarce region to obtain the maximum benefit; when the benefits from water resources development are large, the equal allocation mode can be adopted in severely water-scarce region to obtain the maximum benefit.

## 4 Discussion

The water resource allocation strategies for the Middle East Two Rivers Basin involve an interactive and balanced relationship among the equal allocation, demand priority, and negotiated allocation modes. Although these models differ conceptually and in implementation, integrating them in practice is often necessary to address the actual situation and satisfy all parties' interests. First, the relationship between the three models is explored further below. The equal allocation approach prioritizes fairness, ensuring every country or region has identical basic rights at the discussion's outset. The demand priority mode emphasizes efficiency by allocating resources based on the actual needs and urgency for all parties involved. A natural tension may exist between these two models: fairness could necessitate equal water distribution, whereas efficiency might result in some countries or regions receiving more due to greater demand. To balance this tension during implementation, consultation and negotiation must ensure fair and efficient water distribution. Second, the relationship between the demand priority allocation mode and the negotiated allocation mode. Under the demand priority model, more powerful countries might claim more resources due to higher demand, while weaker countries could receive less due to their lower resource demands. The consultative model provides a platform and mechanism for participants to engage in dialogue and consultation on demands, priorities and sustainable use. Consultation helps balance demand with sustainability, addressing current needs of all parties and guarding against over-exploitation of resources. Third, the relationship between the equal and consultative models. The equal model emphasizes that all parties involved should have the same rights and status. The consultative model provides a practical way to achieve this goal. Consultation allows all parties to express their priorities, needs and commitment to the sustainable management of water resources, which helps build mutual trust and cooperation. The result of consultation may not be complete mathematical equality, but it can be relatively fair based on mutual interests and long-term sustainable goals. Taken together, the three models aim to reach a water resources allocation scheme acceptable to all parties, which requires a combination of equity, efficiency and sustainability. This complex process requires dynamic mediation and continuous consultation efforts by all parties involved. Practically, the equal-sharing model initiates discussions; the demand priority model pinpoints regions needing more resources; and the negotiated-sharing model promotes sustainability, equity, and mutual benefits. When used effectively, these three models complement each other and can create a sensible water resource allocation strategy that addresses the entire basin's requirements and limitations.

According to Conclusion 2, the demand priority allocation mode allocates fewer water resources to an area as the emphasized water shortage degree increases. This is different from the conclusion of Calvete et al. [11]. Calvete et al. [11] proposed that managers prioritized water allocation to users focusing on economic strategies at lower hierarchical levels. Calvete et al. [11] concluded water allocation was hierarchical, in contrast to this article's focus on water shortage degree. Conclusion 2 is caused by the following reasons. Under the demand priority allocation mode, the greater the water shortage degree emphasized by the allocation method, the less water resources are allocated to an area. The demand priority allocation mode allocates water based on an area's economic, social, or environmental benefits. Under this mode, areas or industries with higher efficiency demands are usually given priority to maximize the overall efficiency. If the water shortage degree of an area is larger, it means that the demand for water resources in this area is more urgent and urgent. However, if an area's economic or social benefits are low, or other areas offer greater benefits, its water allocation may be reduced under this mode. Thus, this mode should balance the benefits and needs of different regions to maximize overall benefits. It necessitates a thorough assessment of regional benefits and a sound water allocation strategy. Simultaneously, considering sustainable resource use and environmental protection is crucial to ensure reasonable water allocation and support sustainable development.

According to Conclusions 1 and 3, the greater the impact of unit-level mining on the degree of water shortage, the smaller the amount of water resources allocated to an area. This is different from the study of Hung and Yang [31]. Hung and Yang [31] believed that if the drought lasts, agents can learn and adjust their demands. This article focuses on the allocation perspective, whereas Hung and Yang [31] consider the demand perspective. The rationale behind Conclusions 1 and 3 is explained as follows. The negotiated allocation mode will allocate water resources according to the needs and interests of all parties, and the unit-level mining directly reflects the degree of resources utilization. A high level of unit-level mining reduces the allocation of water resources to an area, as it indicates a need to satisfy a broader range of stakeholder demands. In the negotiated allocation mode, greater resource scarcity leads to heightened and more competitive demand for water resources among stakeholders. High unit-level mining, indicative of intensive resource utilization, may result in a scarce supply and subsequently limit the water resources available for allocation. Therefore, in the negotiated allocation mode, the needs and interests of all parties should be balanced to ensure the reasonable allocation and utilization of resources. Sustainable development strategies that prioritize resource conservation and environmental protection are necessary to optimize benefits and reduce the impact of water shortages on the region.

According to Conclusion 4, when the cost of developing water resources is small and the benefits of developing water resources are large, the demand priority mode can be adopted in water-scarce areas to obtain the maximum benefit. This is different from the study of Tajziehchi et al. [32], who upgraded the HECAM model to HECAM II and concluded how to achieve long-term plans through costs and benefits, thus mitigating social and environmental problems. While this article addresses social issues and introduces models, its primary focus is on the allocation of water resources. Conclusion 4 is mainly caused by the following reasons. The demand priority mode allocates scarce water resources to those with essential needs, chiefly for drinking water, sanitation, and life-sustaining purposes. Adopting the demand priority mode ensures that residents in water-scarce regions have their basic water needs fulfilled. Allocating water to the neediest populations guarantees better health and quality of life. This mode ensures access to reliable, safe drinking water via established supply networks and sustainable facility operations. However, the demand priority mode also faces some challenges. First, it may overlook other water resources’ potential, possibly hindering development elsewhere. Additionally, it demands sensible policies and management to ensure equitable resource allocation and sustainability, preventing misuse or waste. In summary, the demand priority approach is beneficial for water-scarce areas when development costs are low and benefits high, ensuring basic water needs are met and fostering sustainable development.

Conclusion 5 suggests adopting a negotiated allocation mode in water-scarce areas when developing water resources is costly to maximize benefits. This contrasts with Sheikh et al.'s [33] findings. Sheikh et al. [33] considered micro-turbines as a very feasible option to solve the transmission problem in remote areas. They addressed the cost issue of developing water resources through technical means. However, this article solved the problem of water resources allocation from the perspective of allocation mode. The rationale behind Conclusion 5 includes the following. The negotiated allocation mode entails allocating scarce water resources to various stakeholders through discussions, ensuring fair and reasonable sharing. This mode can improve the overall efficiency by formulating a reasonable water resources allocation plan to balance the needs and interests of all parties. A key benefit is its potential to prevent overdevelopment and wastage of water resources, thereby enhancing resource efficiency. Negotiation ensures both fair allocation and effective utilization of water resources, helping avoid disputes and waste. Additionally, it fosters cooperation and mutual benefits among all stakeholders, reducing potential conflicts. However, the negotiated allocation mode also faces some challenges. Firstly, the need for extensive communication can hinder agreement, especially with information asymmetry or low cooperation willingness. Secondly, it requires establishing mechanisms to oversee and enforce the agreement, guaranteeing fair resource allocation and use [24]. In conclusion, despite high development costs, the negotiated allocation mode enables water-scarce areas to maximize benefits, promoting sustainable water resource utilization and management.

According to Conclusion 6, when the benefits of water resources development are greater, the average allocation mode can be adopted in severely water-scarce areas to obtain the maximum benefit. This is different from the research of Chinnasamy et al. [34]. Chinnasamy et al. [34] found that enhancing water infrastructure in India's arid regions bolstered water resources, boosted vegetation, mitigated climate change risks, and, consequently, elevated the socio-economic status of marginalized farmers. Their research focused on specific measures to achieve greater benefits. Whereas this study uses the allocation mode as its lens. The results of this article are mainly caused by the following reasons. If in the development of water resources in the Two River Basin, some areas can obtain greater benefits, then the average allocation mode may indeed be adopted in severely water-scarce areas to obtain certain benefits. The average allocation mode ensures equitable water distribution, allowing every area to receive a consistent supply. This method can alleviate water scarcity in affected areas and satisfy basic water requirements.

This article can provide the following guidance for the actual allocation of water resources in the Tigris-Euphrates River basin. First, it assesses the gap between ideal outcomes and reality across various allocation models, supporting the search for policy tools and mechanisms to bridge this gap. This reconciles expectations with practical water allocation strategies. Second, it anticipates future conflicts and cooperation opportunities. It estimates potential conflicts and overlooked demands under different scenarios, suggesting entry points for cooperation, particularly in extreme weather conditions like droughts or floods. Third, it considers fairness and effectiveness. It offers theoretical and quantitative support to balance fairness and benefits. Assessing the impact of various allocation schemes, it identifies solutions that are fair and effective. The paper aims to develop a feasible and acceptable water resources management framework to reduce tensions, foster cooperation, and enhance efficiency in the basin. The integrated analysis of these models enables countries and regions to develop forward-looking, inclusive, and flexible water policies, supporting the basin's sustainable development and social stability.

## 5 Conclusion

The water resource scarcity in the Middle East's Two River Basin is significant, necessitating a rational allocation approach to optimize economic and social benefits. In this article, the region of the Two River Basin is divided into water shortage area and severe water shortage area. At the same time, three water resources allocation modes are proposed: equal allocation, demand priority or negotiated allocation. Considering the continuous change of decisions in water shortage area and severe water shortage area over time, the article construct a difference game model of the three models. The results show that when the cost of developing water resources is small and the revenue obtained from developing water resources is large, the water-scarce region can obtain the maximum benefit by adopting the demand priority mode. Otherwise, the water-scarce region can obtain the maximum benefit by adopting the negotiated allocation mode. The conclusions of this paper can provide guidance for the actual allocation of water resources in the Tigris-Euphrates River basin. They can help countries and regions in the two Rivers develop more forward-looking, inclusive and flexible water policies that support sustainable development and social stability across the basin.

The research of this article can also be extended. For example, it is assumed that average allocation will lead to inadequate water resources allocation in severe water scarcity areas; allocation according to demand will increase the benefits of the two areas, but will aggravate water resources shortage; in the negotiated allocation mode, the severe water scarcity area needs to pay a certain fee to the water scarcity area. In future research, it can be assumed that with the further shortage of water resources, average allocation will lead to inadequate water resources allocation in water scarcity areas and severe water scarcity areas; with the development of seawater purification technology, allocation according to demand will increase the benefits of the two areas, but will not aggravate water resources shortage; in the negotiated allocation mode, the severe water scarcity area does not need to pay a certain fee to the water scarcity area, etc., for further research. Meanwhile, some blanks in the research can also be solved in future research. First, it is necessary to determine the specific standards adopted in water resources allocation modes in water scarcity areas and severe water scarcity areas under different conditions. Second, the results of water resources allocation should be transformed into practical policy recommendations for reference by water scarcity areas and severe water scarcity areas. Third, in the process of water resources allocation in different areas, water scarcity areas and severe water scarcity areas should determine the order of action of relevant research, rather than taking action at the same time.

## Data availability statement

No data was used for the research described in the article.

## CRediT authorship contribution statement

**Yuntao Bai:** Resources, Project administration, Methodology, Investigation, Data curation. **Lan Wang:** Investigation, Funding acquisition, Conceptualization. **Ruidi Hu:** Methodology, Data curation. **Delong Li:** Resources, Methodology.

## Declaration of competing interest

The authors declare that they have no known competing financial interests or personal relationships that could have appeared to influence the work reported in this paper.

## Appendix 1

Take the derivatives of *G*_*A*1_ with respect to (13), and take the derivatives of *G*_*A*2_ with respect to (14), and set them equal to zero, the article can get:

(43) $$G_{A1}^{*}\left(t\right)=\frac{b_{1}-\frac{\partial V_{A1}}{\partial x_{A1}}f_{1}}{c_{1}}$$

(44) $$G_{A2}^{*}\left(t\right)=\frac{b_{2}+a_{2}-\frac{\partial V_{A2}}{\partial x_{A2}}f_{2}}{c_{2}}$$

Substituting (43) into (13) and substituting (44) into (14), the article can get:

(45) $$\rho V_{A1}=b_{1}\frac{b_{1}-\frac{\partial V_{A1}}{\partial x_{A1}}f_{1}}{c_{1}}-\frac{c_{1}}{2}{\left(\frac{b_{1}-\frac{\partial V_{A1}}{\partial x_{A1}}f_{1}}{c_{1}}\right)}^{2}+lx_{A1}\left(t\right)+\frac{\partial V_{A1}}{\partial x_{A1}}\left[-f_{1}\frac{b_{1}-\frac{\partial V_{A1}}{\partial x_{A1}}f_{1}}{c_{1}}+\delta x_{A1}\left(t\right)\right]$$

(46) $$\rho V_{A2}=b_{2}\frac{b_{2}+a_{2}-\frac{\partial V_{A2}}{\partial x_{A2}}f_{2}}{c_{2}}-\frac{c_{2}}{2}{\left(\frac{b_{2}+a_{2}-\frac{\partial V_{A2}}{\partial x_{A2}}f_{2}}{c_{2}}\right)}^{2}-\left(q_{I}-\frac{b_{2}+a_{2}-\frac{\partial V_{A2}}{\partial x_{A2}}f_{2}}{c_{2}}\right)a_{2}+lx_{A2}\left(t\right)+\frac{\partial V_{A2}}{\partial x_{A2}}\left[-f_{2}\frac{b_{2}+a_{2}-\frac{\partial V_{A2}}{\partial x_{A2}}f_{2}}{c_{2}}+\delta x_{A2}\left(t\right)\right]$$

Let $V_{A1}^{*}=k_{1}x_{A1}+k_{2}$, $V_{A2}^{*}=k_{3}x_{A2}+k_{4}$, wherein, *k*_1_, *k*_2_, *k*_3_ and *k*_4_ are all constants. The parameters of the optimal social welfare function can be obtained by calculation as follows:

(47) $$\left\{ \begin{array}{c} k_{1}=\frac{l}{\rho -\delta }\\ k_{2}=\frac{1}{\rho }b_{1}\frac{b_{1}-\frac{l}{\rho -\delta }f_{1}}{c_{1}}-\frac{c_{1}}{2}\frac{1}{\rho }{\left(\frac{b_{1}-\frac{l}{\rho -\delta }f_{1}}{c_{1}}\right)}^{2}-\frac{1}{\rho }\frac{l}{\rho -\delta }f_{1}\frac{b_{1}-\frac{l}{\rho -\delta }f_{1}}{c_{1}} \end{array}\right.$$

(48) $$\left\{ \begin{array}{c} k_{3}=\frac{l}{\rho -\delta }\\ k_{4}=\frac{1}{\rho }b_{2}\frac{b_{2}+a_{2}-\frac{\partial V_{A2}}{\partial x_{A2}}f_{2}}{c_{2}}-\frac{c_{2}}{2}\frac{1}{\rho }{\left(\frac{b_{2}+a_{2}-\frac{\partial V_{A2}}{\partial x_{A2}}f_{2}}{c_{2}}\right)}^{2}-\frac{1}{\rho }\left(q_{I}-\frac{b_{2}+a_{2}-\frac{\partial V_{A2}}{\partial x_{A2}}f_{2}}{c_{2}}\right)a_{2}\\ -\frac{\partial V_{A2}}{\partial x_{A2}}\frac{1}{\rho }f_{2}\frac{b_{2}+a_{2}-\frac{\partial V_{A2}}{\partial x_{A2}}f_{2}}{c_{2}} \end{array}\right.$$

Therefore, it can be concluded that:

(49) $$V_{A1}^{*}=\frac{l}{\rho -\delta }x_{A1}+\frac{1}{\rho }b_{1}\frac{b_{1}-\frac{l}{\rho -\delta }f_{1}}{c_{1}}-\frac{c_{1}}{2}\frac{1}{\rho }{\left(\frac{b_{1}-\frac{l}{\rho -\delta }f_{1}}{c_{1}}\right)}^{2}-\frac{1}{\rho }\frac{l}{\rho -\delta }f_{1}\frac{b_{1}-\frac{l}{\rho -\delta }f_{1}}{c_{1}}$$

(50) $$V_{A2}^{*}=\frac{l}{\rho -\delta }x_{A2}+\frac{1}{\rho }b_{2}\frac{b_{2}+a_{2}-\frac{\partial V_{A2}}{\partial x_{A2}}f_{2}}{c_{2}}-\frac{c_{2}}{2}\frac{1}{\rho }{\left(\frac{b_{2}+a_{2}-\frac{\partial V_{A2}}{\partial x_{A2}}f_{2}}{c_{2}}\right)}^{2}-\frac{1}{\rho }\left(q_{I}-\frac{b_{2}+a_{2}-\frac{\partial V_{A2}}{\partial x_{A2}}f_{2}}{c_{2}}\right)a_{2}-\frac{\partial V_{A2}}{\partial x_{A2}}\frac{1}{\rho }f_{2}\frac{b_{2}+a_{2}-\frac{\partial V_{A2}}{\partial x_{A2}}f_{2}}{c_{2}}$$

in this case,

(51) $$G_{A1}^{*}\left(t\right)=\frac{b_{1}-\frac{l}{\rho -\delta }f_{1}}{c_{1}}$$

(52) $$G_{A2}^{*}\left(t\right)=\frac{b_{2}+a_{2}-\frac{l}{\rho -\delta }f_{2}}{c_{2}}$$

## Appendix 2

Take the derivatives of *G*_*R*1_ with respect to (15), and take the derivatives of *G*_*R*2_ with respect to (16), and set them equal to zero, the article can get:

(53) $$G_{R1}^{*}\left(t\right)=\frac{b_{1}+b_{R}-\frac{\partial V_{R1}}{\partial x_{R1}}\left(f_{1}+f\right)}{c_{1}}$$

(54) $$G_{R2}^{*}\left(t\right)=\frac{b_{2}+b_{R}-\frac{\partial V_{R2}}{\partial x_{R2}}\left(f_{2}+f\right)}{c_{2}}$$

Substituting (53) into (15) and substituting (54) into (16), the article can get:

(55) $$\rho V_{R1}=\left(b_{1}+b_{R}\right)\frac{b_{1}+b_{R}-\frac{\partial V_{R1}}{\partial x_{R1}}\left(f_{1}+f\right)}{c_{1}}-\frac{c_{1}}{2}{\left[\frac{b_{1}+b_{R}-\frac{\partial V_{R1}}{\partial x_{R1}}\left(f_{1}+f\right)}{c_{1}}\right]}^{2}+lx_{A1}\left(t\right)+\frac{\partial V_{R1}}{\partial x_{R1}}\left[-\left(f+f_{1}\right)\frac{b_{1}+b_{R}-\frac{\partial V_{R1}}{\partial x_{R1}}\left(f_{1}+f\right)}{c_{1}}+\delta x_{R1}\left(t\right)\right]$$

(56) $$\rho V_{R2}=\left(b_{2}+b_{R}\right)\frac{b_{2}+b_{R}-\frac{\partial V_{R2}}{\partial x_{R2}}\left(f_{2}+f\right)}{c_{2}}-\frac{c_{2}}{2}{\left[\frac{b_{2}+b_{R}-\frac{\partial V_{R2}}{\partial x_{R2}}\left(f_{2}+f\right)}{c_{2}}\right]}^{2}+lx_{A2}\left(t\right)+\frac{\partial V_{R2}}{\partial x_{R2}}\left[-\left(f+f_{2}\right)\frac{b_{2}+b_{R}-\frac{\partial V_{R2}}{\partial x_{R2}}\left(f_{2}+f\right)}{c_{2}}+\delta x_{R2}\left(t\right)\right]$$

Let $V_{R1}^{*}=k_{5}x_{R1}+k_{6}$, $V_{R2}^{*}=k_{7}x_{R2}+k_{8}$, wherein, *k*_5_, *k*_6_, *k*_7_ and *k*_8_ are all constants. The parameters of the optimal social welfare function can be obtained by calculation as follows:

(57) $$\left\{ \begin{array}{c} k_{5}=\frac{l}{\rho -\delta }\\ k_{6}=\frac{1}{\rho }\left(b_{1}+b_{R}\right)\frac{b_{1}+b_{R}-\frac{l}{\rho -\delta }\left(f_{1}+f\right)}{c_{1}}-\frac{c_{1}}{2}\frac{1}{\rho }{\left[\frac{b_{1}+b_{R}-\frac{l}{\rho -\delta }\left(f_{1}+f\right)}{c_{1}}\right]}^{2}\\ -\frac{l}{\rho -\delta }\frac{1}{\rho }\left(f+f_{1}\right)\frac{b_{1}+b_{R}-\frac{l}{\rho -\delta }\left(f_{1}+f\right)}{c_{1}} \end{array}\right.$$

(58) $$\left\{ \begin{array}{c} k_{7}=\frac{l}{\rho -\delta }\\ k_{8}=\frac{1}{\rho }\left(b_{2}+b_{R}\right)\frac{b_{2}+b_{R}-\frac{l}{\rho -\delta }\left(f_{2}+f\right)}{c_{2}}-\frac{c_{2}}{2}\frac{1}{\rho }{\left[\frac{b_{2}+b_{R}-\frac{l}{\rho -\delta }\left(f_{2}+f\right)}{c_{2}}\right]}^{2}\\ -\frac{l}{\rho -\delta }\frac{1}{\rho }\left(f+f_{2}\right)\frac{b_{2}+b_{R}-\frac{l}{\rho -\delta }\left(f_{2}+f\right)}{c_{2}} \end{array}\right.$$

Therefore, it can be concluded that:

(59) $$V_{R1}^{*}=\frac{l}{\rho -\delta }x_{R1}+\frac{1}{\rho }\left(b_{1}+b_{R}\right)\frac{b_{1}+b_{R}-\frac{l}{\rho -\delta }\left(f_{1}+f\right)}{c_{1}}-\frac{c_{1}}{2}\frac{1}{\rho }{\left[\frac{b_{1}+b_{R}-\frac{l}{\rho -\delta }\left(f_{1}+f\right)}{c_{1}}\right]}^{2}-\frac{l}{\rho -\delta }\frac{1}{\rho }\left(f+f_{1}\right)\frac{b_{1}+b_{R}-\frac{l}{\rho -\delta }\left(f_{1}+f\right)}{c_{1}}$$

(60) $$V_{R2}^{*}=\frac{l}{\rho -\delta }x_{R2}+\frac{1}{\rho }\left(b_{2}+b_{R}\right)\frac{b_{2}+b_{R}-\frac{l}{\rho -\delta }\left(f_{2}+f\right)}{c_{2}}-\frac{c_{2}}{2}\frac{1}{\rho }{\left[\frac{b_{2}+b_{R}-\frac{l}{\rho -\delta }\left(f_{2}+f\right)}{c_{2}}\right]}^{2}-\frac{l}{\rho -\delta }\frac{1}{\rho }\left(f+f_{2}\right)\frac{b_{2}+b_{R}-\frac{l}{\rho -\delta }\left(f_{2}+f\right)}{c_{2}}$$

in this case,

(61) $$G_{R1}^{*}\left(t\right)=\frac{b_{1}+b_{R}-\frac{l}{\rho -\delta }\left(f_{1}+f\right)}{c_{1}}$$

(62) $$G_{R2}^{*}\left(t\right)=\frac{b_{2}+b_{R}-\frac{l}{\rho -\delta }\left(f_{2}+f\right)}{c_{2}}$$

## Appendix 3

Take the derivatives of *G*_*N*1_ with respect to (17), and take the derivatives of *G*_*N*2_ with respect to (18), and set them equal to zero, the article can get:

(63) $$G_{N1}^{*}\left(t\right)=\frac{b_{1}-\frac{\partial V_{N1}}{\partial x_{N1}}f_{1}}{c_{1}}$$

(64) $$G_{N2}^{*}\left(t\right)=\frac{b_{2}-\frac{\partial V_{N2}}{\partial x_{N2}}f_{2}}{c_{2}}$$

Substituting (63) into (17) and substituting (64) into (18), the article can get:

(65) $$\rho V_{N1}=b_{1}\frac{b_{1}-\frac{\partial V_{N1}}{\partial x_{N1}}f_{1}}{c_{1}}-\frac{c_{1}}{2}{\left(\frac{b_{1}-\frac{\partial V_{N1}}{\partial x_{N1}}f_{1}}{c_{1}}\right)}^{2}+F_{N}+lx_{N1}\left(t\right)+\frac{\partial V_{N1}}{\partial x_{N1}}\left[-f_{1}\frac{b_{1}-\frac{\partial V_{N1}}{\partial x_{N1}}f_{1}}{c_{1}}+\delta x_{N1}\left(t\right)\right]$$

(66) $$\rho V_{N2}=b_{2}\frac{b_{2}-\frac{\partial V_{N2}}{\partial x_{N2}}f_{2}}{c_{2}}-\frac{c_{2}}{2}{\left(\frac{b_{2}-\frac{\partial V_{N2}}{\partial x_{N2}}f_{2}}{c_{2}}\right)}^{2}-F_{N}+lx_{N2}\left(t\right)+\frac{\partial V_{N2}}{\partial x_{N2}}\left[-f_{2}\frac{b_{2}-\frac{\partial V_{N2}}{\partial x_{N2}}f_{2}}{c_{2}}+\delta x_{N2}\left(t\right)\right]$$

Let $V_{N1}^{*}=k_{9}x_{N1}+k_{10}$, $V_{N2}^{*}=k_{11}x_{N2}+k_{12}$, wherein, *k*_9_, *k*_10_, *k*_11_ and *k*_12_ are all constants. The parameters of the optimal social welfare function can be obtained by calculation as follows:

(67) $$\left\{ \begin{array}{c} k_{9}=\frac{l}{\rho -\delta }\\ k_{10}=\frac{1}{\rho }b_{1}\frac{b_{1}-\frac{l}{\rho -\delta }f_{1}}{c_{1}}-\frac{c_{1}}{2}\frac{1}{\rho }{\left(\frac{b_{1}-\frac{l}{\rho -\delta }f_{1}}{c_{1}}\right)}^{2}+\frac{1}{\rho }F_{N}-\frac{1}{\rho }\frac{l}{\rho -\delta }f_{1}\frac{b_{1}-\frac{l}{\rho -\delta }f_{1}}{c_{1}} \end{array}\right.$$

(68) $$\left\{ \begin{array}{c} k_{11}=\frac{l}{\rho -\delta }\\ k_{12}=\frac{1}{\rho }b_{2}\frac{b_{2}-\frac{l}{\rho -\delta }f_{2}}{c_{2}}-\frac{c_{2}}{2}\frac{1}{\rho }{\left(\frac{b_{2}-\frac{l}{\rho -\delta }f_{2}}{c_{2}}\right)}^{2}-\frac{1}{\rho }F_{N}-\frac{l}{\rho -\delta }\frac{1}{\rho }f_{2}\frac{b_{2}-\frac{l}{\rho -\delta }f_{2}}{c_{2}} \end{array}\right.$$

Therefore, it can be concluded that:

(69) $$V_{N1}^{*}=\frac{l}{\rho -\delta }x_{N1}+\frac{1}{\rho }b_{1}\frac{b_{1}-\frac{l}{\rho -\delta }f_{1}}{c_{1}}-\frac{c_{1}}{2}\frac{1}{\rho }{\left(\frac{b_{1}-\frac{l}{\rho -\delta }f_{1}}{c_{1}}\right)}^{2}+\frac{1}{\rho }F_{N}-\frac{1}{\rho }\frac{l}{\rho -\delta }f_{1}\frac{b_{1}-\frac{l}{\rho -\delta }f_{1}}{c_{1}}$$

(70) $$V_{N2}^{*}=\frac{l}{\rho -\delta }x_{N2}+\frac{1}{\rho }b_{2}\frac{b_{2}-\frac{l}{\rho -\delta }f_{2}}{c_{2}}-\frac{c_{2}}{2}\frac{1}{\rho }{\left(\frac{b_{2}-\frac{l}{\rho -\delta }f_{2}}{c_{2}}\right)}^{2}-\frac{1}{\rho }F_{N}-\frac{l}{\rho -\delta }\frac{1}{\rho }f_{2}\frac{b_{2}-\frac{l}{\rho -\delta }f_{2}}{c_{2}}$$

in this case,

(71) $$G_{N1}^{*}\left(t\right)=\frac{b_{1}-\frac{l}{\rho -\delta }f_{1}}{c_{1}}$$

(72) $$G_{N2}^{*}\left(t\right)=\frac{b_{2}-\frac{l}{\rho -\delta }f_{2}}{c_{2}}$$
